# Phytochemical Profile, Mineral Content, and Bioactive Compounds in Leaves of Seed-Propagated Artichoke Hybrid Cultivars

**DOI:** 10.3390/molecules25173795

**Published:** 2020-08-20

**Authors:** Gabriele Rocchetti, Luigi Lucini, Giandomenico Corrado, Giuseppe Colla, Mariateresa Cardarelli, Stefania De Pascale, Youssef Rouphael

**Affiliations:** 1 Department for Sustainable Food Process, Research Centre for Nutrigenomics and Proteomics, University Cattolica del Sacro Cuore, 29122 Piacenza, Italy; gabriele.rocchetti@unicatt.it; 2 Department of Agricultural Sciences, University of Naples Federico II, 80055 Portici, Italy; giandomenico.corrado@unina.it (G.C.); depascal@unina.it (S.D.P.); 3 Department of Agriculture and Forest Sciences, University of Tuscia, 01100 Viterbo, Italy; giucolla@unitus.it; 4 CREA-Research Centre for Vegetable and Ornamental Crops, 84098 Pontecagnano Faiano, Italy; mteresa.cardarelli@crea.gov.it

**Keywords:** *Cynara cardunculus*, UHPLC-QTOF, polyphenols, sesquiterpenoids, metabolomics, multivariate statistics

## Abstract

The globe artichoke (*Cynara cardunculus* L. subsp. *Scolymus* (L.) Hegi) is a multi-year species rich in various classes of phytochemicals with known nutritional and pharmacological properties, such as polyphenols, sesquiterpene lactones, and terpenoids. Over the last decade, hybrids cultivars are transforming the artichoke market for their higher uniformity and stability over the traditional landraces, further increasing the potential of the artichoke as a source of commercial extracts and bioactive molecules. Our aim was to investigate the mineral and phytochemical profiles of leaves from seven seed-propagated hybrids by using an untargeted metabolomic approach based on ultra-high-pressure liquid chromatography coupled with quadrupole-time-of-flight mass spectrometry. Metabolomics identified several compounds in the tested varieties, namely 98 polyphenols, 123 sesquiterpene lactones, and 221 other metabolites. The phenolic content ranged from 3.01 mg Eq./g fw (for ‘Opera’) to 4.71 mg Eq./g fw (for ‘Opal’). Sesquiterpene lactones were, on average, 2.11 mg Eq./g fw. Multivariate statistics (HCA, PCA and OPLS-DA) highlighted the main metabolomics differences among cultivars, which weakly correlated with their agronomic classification. The seven cultivars showed distinctive metabolomics profiles, with ‘Opal’ and ‘Istar’ being the most valuable hybrids. The 3-hydroxyphenyl-valeric acid (a medium-chain fatty acid) and the 6-Gingesulfonic acid (a methoxyphenol) were the most discriminant markers. Our findings illustrated the quantitative and qualitative variation of several classes of phytochemicals in seed-propagated artichoke cultivars and allowed identifying distinctive metabolic signatures for both phenolic compounds and sesquiterpene lactones. This work supports the exploitation of the artichoke leaves from hybrid cultivars as a rich source of bioactive phytochemicals.

## 1. Introduction

The Asteraceae family, previously known as Compositeae, comprises different economically important crops with nutraceutical and pharmacological properties, such as the artichoke [1]. According to the Compositae Global Database (https://www.compositae.org/), the (globe) artichoke is classified as *Cynara cardunculus* subspecies *scolymus* (L.) Hegi, one of the various interfertile cultivated groups of this species.

Epidemiological and pharmacological studies have demonstrated the health-promoting effects of globe artichoke extracts, including hypolipidemic, hepatoprotective, anti-obesity, anti-tumor, antioxidant, and anti-inflammatory activity [1]. For this reason, in addition to the culinary use of the budding flower-head, the abundant leaf biomass of this species has gained importance as a raw material for the production of nutraceuticals, food preservatives, drugs and cosmetics [2,3]. The artichoke is well known as a valuable source of various bioactive compounds (such as polyphenols), soluble dietary fiber (e.g., inulin), vitamins, and minerals [1,4]. Specifically, leaf extracts are rich in phenolic acids (e.g., isomers of caffeoylquinic acid), flavones (such as glycosidic forms of luteolin and apigenin), anthocyanins (mainly glycosidic derivatives of cyanidin), and lower-molecular-weight phenolics [5,6]. Sesquiterpene lactones are also an important class of bioactive compounds that characterize artichoke and its beneficial properties. For instance, cynaropicrin, which is mainly present in leaves, has a range of pharmacologic properties, including antihyperlipidemic, antitrypanosomal, antimalarial, anti-inflammatory, antifeedant, antispasmodic, antiphotoaging, and antitumoral action, as well as being a main factor activating bitter sensory receptors [2]. The quantity of polyphenols and sesquiterpene lactones, like other metabolites, depends on preharvest factors such as the genotype, the growth condition, the agronomic technique and the phenological stage [7,8,9]. Previous studies indicated the presence of a significant variation in the profile of bioactive compounds among artichoke varieties, hinting to the existence of considerable genetic variability of secondary metabolites under controlled conditions [10,11].

Since the last two decades, the artichoke cultivation has seen a progressive success of seed-propagated varieties [7,10,11]. This diffusion has also been favored by the growing demand for highly uniform genotypes capable of producing larger biomass also in advanced soilless cultivation systems, such as the high-planting density floating raft hydroponics system [7,12]. In addition to known advantages of soilless cultivation (e.g., mitigation of soil pathogens and abiotic stress; reduced labor; easy of harvest; cleanness of the leaves), specific benefits of the floating raft system are the possibility to increase artichoke density without weed control pressure, as well as the number of harvests. Moreover, hydroponics provides the opportunity to alter the secondary metabolism of plants and the sensory/functional quality of the product through controlled positive stress applied by way of nutrient solution composition [8,12,13,14,15].

Compared to other plants, the industrial processing of artichoke has been hindered by some of the typical features of an agamically propagated, multi-year crop. These include the asynchronous maturity (which requires a multiple-picking harvest), the size and vigor variability of the starting material (e.g., offshoots, ‘ovoli’ or rhizome parts), the intra-landraces genetic variability, and the uncertain phytosanitary condition of the clones. The introduction of distinct, uniform, and stable seed-propagated hybrid varieties has greatly supported the industrial transformation of artichoke, typically for the commercialization of the canned flower buds. Moreover, modern varieties are usually characterized by an increased number of leaves, mainly because this trait increases the head mass and indirectly, its tenderness and compactness. In addition, hybrid cultivars more effectively profit from standardized and controlled growing conditions (e.g., protected and/or soilless systems). Finally, artichoke hybrids are more amenable to high-density planting because of earliness of production. All these features have revitalized the interest in artichoke leaves as a source of bioactive extracts. Nonetheless, a thorough description of functionally relevant molecules and their variation in modern hybrid cultivars is currently not available.

The aim of this work was to investigate the metabolomics profile and the mineral composition of modern hybrid cultivars growing in an advanced cultivation system under a high density of planting. Our interest was to understand the phytochemical profile and the range of variation of leaf extracts from modern artichoke varieties. Moreover, emphasis was also given to the identification of cultivar-specific metabolomic signatures, which can be helpful in selecting appropriate material for selected functional components or the production of functional extracts.

## 2. Results and Discussion

### 2.1. Leaf Biomass Production and Mineral Profile

**Figure 1:**
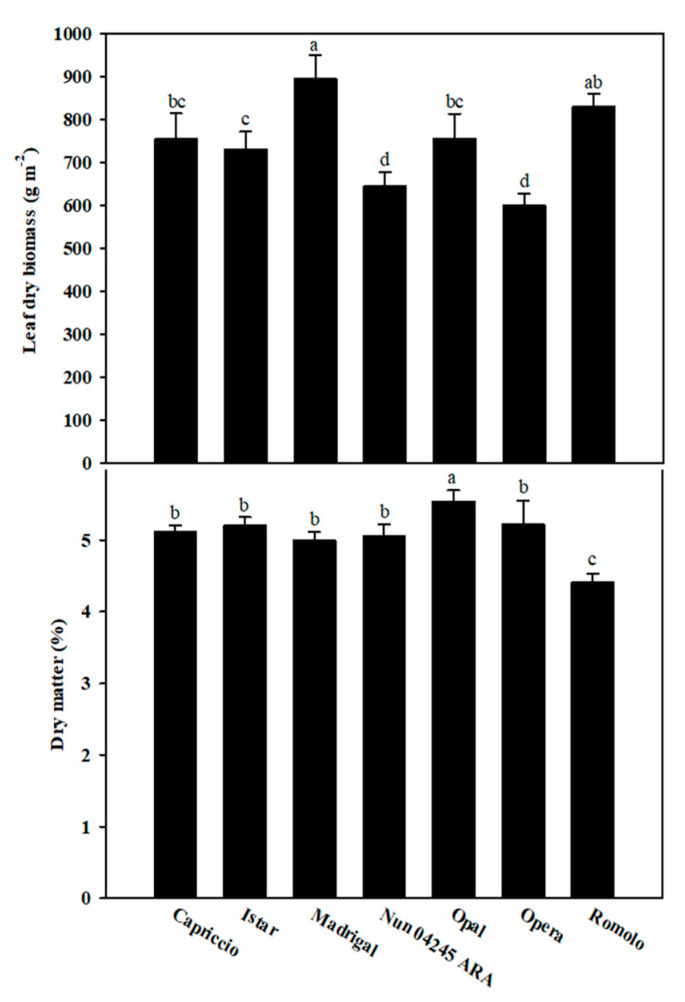
Leaf dry biomass and leaf dry matter content of seven artichoke cultivars grown in a floating raft culture. Each bar represents the mean ± standard deviation (*n* = 3). For each bar, different letters indicate statistically different groups (*p* < 0.05, Duncan’s post-hoc test following ANOVA; *n* = 3).

The leaf dry biomass of artichoke seed-propagated hybrids ranged from 600 to 896 g m^−2^ and significant differences were recorded between cultivars (Figure 1).

The lower biomass (<650 g m^−2^) was produced by ‘Nun 04245 ARA’ and ‘Opera’; the highest by ‘Madrigal’ and ‘Romolo’, the two late varieties (Figure 1). The average leaf biomass in the current study (745 g m^−2^) was lower than that previously reported by Colla et al. [7] on three artichoke cultivars (‘Romolo’, Violetto di Provenza’ and ‘Violetto di Romagna’; avg. 1680 g m^−2^). This variation probably reflects the different plant density (531 vs. 463 plants per square meter), the different growing season (autumn-winter vs. spring-summer), and the length of the cultivation period (62 vs. 100 days after sowing). The mean leaf dry matter of the seven artichoke cultivars was 5.1%, ranging from 4.4% in ‘Romolo’ to 5.5% in ‘Opal’ (Figure 1). The average leaf dry matter was lower than that previously reported (avg. 10.1%) [5]. For leafy vegetables, the products from soilless culture have a lower dry weight, although this does not necessarily result in a lower yield [16].

We also investigated the mineral composition of the leaves because minerals are usually retained in plant extracts and define some of their functional attributes. Specifically, some mineral elements play important roles not only for several enzymes involved in cell metabolism but also for antioxidant systems, having in some instances, a positive role in disease prevention [17]. Potassium was the most abundant macro mineral in leaves (average 76.1 mg g^−1^ dw), followed by nitrogen (average 47.8 mg g^−1^ dw), calcium (average 9.8 mg g^−1^ dw), phosphorus (average 6.5 mg g^−1^ dw), and magnesium (average 3.9 mg g^−1^ dw) (Table 1).

Our findings are in line with those of Colla et al. [7], who reported that potassium was the most abundant macronutrient present in three cultivars of artichoke (‘Romolo’, Violetto di Provenza’ and ‘Violetto di Romagna’) grown in floating raft culture. The high concentration of potassium in leaf tissue of globe artichoke has been reported earlier by Rincon et al. [18] and Pandino et al. [19], likely because potassium is the macronutrient most absorbed and translocated to the shoot by artichoke plants. A significant difference among the seven artichoke cultivars was not observed for nitrogen and calcium concentration in leaf tissue, while the levels of phosphorus, potassium and magnesium revealed significant differences (Table 1). Phosphorous had the higher coefficient of variation (13%), followed by Mg (7%) and K (6%). Overall, the differences in the mineral content in leaves between cultivars were limited, most likely because plants were fed by a rich nutrient solution hence, not prone to mineral deficiency or suboptimal availability. This is also a likely reason of the lack of a significant correlation among the content of the three statistically different minerals (not shown).

### 2.2. Phytochemical Profile of the Different Artichoke Seed Propagated Cultivars

The phytochemical composition of the different artichoke cultivars was investigated by using an untargeted metabolomic approach. Overall, 442 compounds were putatively identified utilizing both the Phenol-Explorer and Food Database. Specifically, we identified 98 polyphenols, 123 sesquiterpene lactones, and 221 compounds classified as other metabolites (including benzenoids, lipid- and amino acid-derivatives). The annotated compounds are presented in Supplementary Table S1, along with the corresponding composite mass spectra (mass and abundances combinations). Among polyphenols, flavonoids (*n* = 53) represented the most abundant class of compounds (including 6 anthocyanins, 6 flavan-3-ols, 9 flavanones, 8 flavonols, 3 isoflavonoids and 18 flavones), followed by phenolic acids (*n* = 14, mainly hydroxycinnamics), lignans (*n* = 13), and other polyphenols (such as alkylphenols and tyrosol derivatives). Tyrosol, gardenin B, caffeic acid ethyl ester, caffeoyl aspartic acid, and 24-Methylcholestanol ferulate (both hydroxycinnamic acids) were among the most abundant compounds. Tyrosol has been described mainly in olives and olive oil and is associated to a wide range of health benefits, such as cardioprotective, antiviral, anti-inflammatory and anticancer activities [20]. Gardenin B, also known as demethyltangeretin or 5-hydroxy-4′,6,7,8-tetramethoxyflavone, is a methoxyflavonoid (or 8-*O*-methylated flavonoid). This compound has been reported to have a cytotoxicity against cancer cell lines (HL-60 and U-937), even stronger than quercetin, probably the best-studied flavonoid [21]. Caffeic acid derivatives are known to possess anti-inflammatory, antioxidant, anti-tumor, and antibacterial activities, and can contribute to the prevention of atherosclerosis and other cardiovascular diseases [22]. The most abundant sesquiterpene lactones were anthemolide B, ixerisoside K, and chlorohyssopifolin B, together with its isomer chlorochrymorin (Supplementary Table S1).

The phytochemical profiles of artichoke were consistent with those reported in previous works [5]. Rouphael and co-authors showed that the phenolic profile of leaf extracts from nineteen cultivars included flavonoids, phenolic acids (mainly hydroxycinnamic acids), tyrosol derivatives, and lignans [5]. Artichoke is very abundant in polyphenols, mainly hydroxycinnamics acid derivatives, which represent a significant fraction of the whole secondary metabolites. In our experimental conditions, hydroxycinnamic acids, such as positional isomers of dicaffeoylquinic acid, were not detected. Their content also depends on morphogenesis, and it was reported that apical, young leaves are a better source of caffeoylquinic acids [23]. In addition, the existence of several isozymes of quinate hydroxycinnamoyl transferase (HQT) in artichoke could potentially explain the possible fluctuations in chlorogenic acid levels in the selected cultivars [24].

By exploiting a semi-quantitative analysis from the UHPLC-QTOF data, the polyphenols and sesquiterpene lactones identified were then quantified according to a representative standard per class and expressed in mg Equivalent (Eq.) g^−1^ fresh weight (fw) (Table 2).

In all the artichoke cultivars, the cumulative phenolic content was larger than that of the sesquiterpene lactones (Table 2). Specifically, the total phenolic content (i.e., the cumulative value of the different phenolic classes) ranged from 3.01 mg Eq. g^−1^ fw (for ‘Opera’) up to 4.71 (for ‘Opal’), while sesquiterpene lactones showed an average content of 2.11 mg Eq. g^−1^ fw. The total phenolic content and the sesquiterpene lactones had very similar coefficient of variations across varieties (15%). Higher differences were present for some specific phenolic classes. The cultivar ‘Istar’ showed the highest cumulative contents of phenyl alcohol and flavonol, being 3.334 and 0.089 mg Eq. g^−1^ fw, respectively. ‘Capriccio’ showed the highest content of flavones. Moreover, flavonols was the phenolic class with the higher variation among cultivars. The higher content of phenolic acids was recorded for the cultivar ‘Madrigal’ and Nun 04245 ARA (on average 0.436 mg Eq. g^−1^ fw). Phenyl alcohols (quantified as tyrosol equivalents) were the most abundant class of polyphenols, representing almost the 60% of the total. Flavones, lignans, and phenolic acids were the second largest contributors to the cumulative phenolic content.

The UHPLC-QTOF analysis also revealed traces of anthocyanins in artichoke leaf extracts, with the highest value in the ‘Istar’ cultivar (Table 2). Previous works also reported that flavones and phenolic acids are the main compounds of artichoke leaf extracts [19,25,26,27,28,29]. Anthocyanins are the main flavonoid class responsible for blue/purple color in many plant tissues and, in particular, for the pigmentation of the globe artichoke bracts. A solid correlation between head color and the quantity of anthocyanins in leaves was not evident. For instance, while the green type ‘Madrigal’ had the lowest value, a similar amount was recorded in hybrids that develop capitula of darker color. In plants, anthocyanins strongly accumulate in response to various stresses and are mainly transcriptionally regulated [30]. Our data imply that the head color classification is not a good predictor of the amount of anthocyanins in leaves. The analyzed artichoke cultivars were rich in flavone equivalents (on average: 0.68 mg Eq. g^−1^ fw), in agreement with previous evidence on this phenolic subclass in artichoke [2]. Flavones are one of the most important flavonoids subgroups [31], present in artichoke leaves mainly as glycosides [2]. These compounds have been intensely studied and characterized for their biological properties, including antioxidant, antiviral, and antibacterial activities [32]. Finally, lignans are another typical chemical class in artichoke leaf extracts and the UHPLC-QTOF revealed several sesamin and matairesinol derivatives (Table S1). A significant difference in lignans was not present among cultivars (Table 2), probably because this subgroup of non-flavonoid polyphenols has usually a structural role in plants. Artichoke leaf extracts had an average of 0.450 mg Eq. g^−1^ fw of lignans, a value higher than most cereals, nuts, vegetables, and legumes, with the notable and well-known exceptions of flax and sesame seeds [33]. Lignans also possess physiological properties as antioxidants, phytoestrogens, antimicrobial, and anti-inflammatory molecules [34,35,36]. Specifically, artichoke leaves displayed a large variation in pinoresinol (PIN), one of the few well-characterized lignan-derived phytoestrogens [37].

### 2.3. Multivariate Discrimination of the Seed-Propagated Artichoke Cultivars and Identification of Marker Compounds

**Figure 2:**
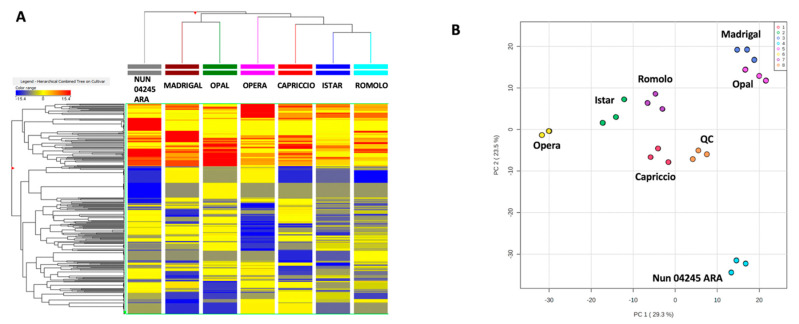
Heat map from unsupervised hierarchical cluster analysis (**A**) and principal component analysis (PCA) score plot (**B**) built considering the phytochemical profile of the different artichoke cultivars. In the PCA score plot, the distribution of Quality Control (QC) sample replicates is also showed.

Considering the differences on phenolics and sesquiterpenes, multivariate statistical approaches were used to analyze the phytochemical similarity and possibly, hidden patterns across the seven cultivars under investigation. The outputs of the explanatory statistics (HCA and PCA) are shown in Figure 2A,B. The heatmap graphically illustrated the limited overlap among the metabolic profiles, with each cultivar presenting a distinct profile from the others. This can be explained considering that our germplasm collection is characterized by varieties that cover the different breeding classes of artichoke (e.g., in relation to the time of maturity as well as the head color and shape). To better organize varieties according to the large phytochemical data set, we performed an HCA on varieties. There was no convincing grouping based on some key shared characteristics of the varieties (e.g., the color of the capitula). For instance, HCA indicated that the most similar varieties were ‘Istar’ and ‘Romolo’. The former is a very early hybrid with an American pedigree, while the latter is a medium-late Romanesco type. In a similar way, the profile of ‘Madrigal’, a late-type with large green heads, was most closely related to the profile of ‘Opal’, an early-type with predominantly violet heads.

To explore the intrinsic variability of the data through dimensionality reduction, we performed also a PCA on single replicates. The first two components explained 52.8% of the total variance. The PCA showed that biological replicates formed clusters, whose dispersion was similar among cultivars. This is an indirect evidence of the genetic uniformity typical of hybrid cultivars, marked especially when plants grow in controlled conditions. Moreover, the PCA provided a graphical representation of the distances between varieties. Cultivars were well distributed mainly through the PC1, with ‘Nun 04245 ARA’ present in a spatially distinct region.

**Figure 3:**
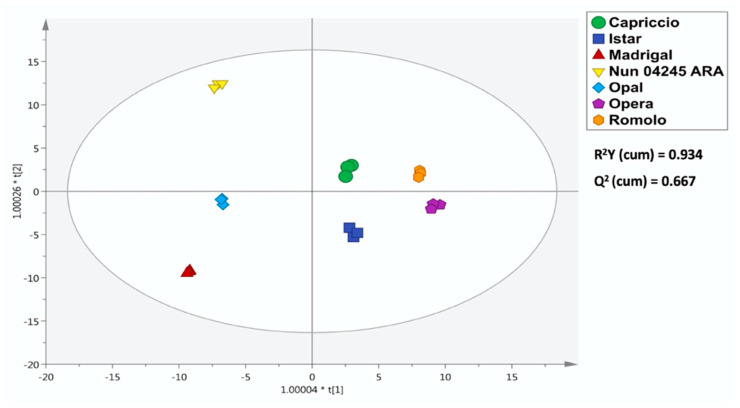
Orthogonal projection to latent structures discriminant analysis (OPLS-DA) built considering the phytochemical profile (from UHPLC-QTOF) of the different artichoke cultivars.

Having characterized the variability and consistency of the metabolomics data, we performed an OPLS-DA to group similar subsets of samples together (Figure 3). The analysis showed that the groups of each replicate were tight and sufficiently separated to draw confidence borders for all the different class models.

Specifically, the model cross-validation parameters of the OPLS-DA model were very high (R^2^Y = 0.934 and Q^2^Y = 0.667), with adequate cross validation parameters (CV-ANOVA—*p* < 0.01) and permutation test cross-validation (N = 200). These model parameters confirmed the robustness of the model to discriminate the profiles of the artichoke cultivars under investigation (Supplementary Table S2). Moreover, the output of Hotelling’s T2 (Supplementary Table S2) indicated that none of the replicates exceeded the 95% (respectively, 99%) confidence limits for the suspected (resp. strong) outliers. To assess the contribution that a compound makes to the OPLS-DA model, we calculated the variables importance in projection (VIP) score for each annotated compound. The VIP score is useful to summarize the discrimination power of individual variables. Table 3 reports the marker compounds with a VIP score higher than 1.1. In addition, the Log Fold-Change (FC) values relative to the cultivar ‘Opal’, the richest source of polyphenols, are reported as supplementary material (Table S3).

Overall, 111 compounds (including potential isomers) mostly contributed to the pattern of the OPLS-DA score plot. The two compounds with the highest VIP scores were the medium-chain fatty acid 3-Hydroxyphenyl-valeric acid (VIP score = 1.43) and the methoxyphenol 6-Gingesulfonic acid (VIP score = 1.38). The 3-Hydroxyphenyl-valeric acid characterized the ‘Capriccio’ (LogFC value = 0.77) and the ‘Romolo’ (LogFC value = 0.26) cultivars. The 6-Gingesulfonic acid was detected in all cultivars, except ‘Istar’ (Table S1). Flavonoids were the most abundant phenolic markers (28 annotated compounds), with flavone derivatives (such as 2-Hydroxynaringenin and eriodictyol) possessing the higher VIP scores (i.e., >1.2). Eriodictyol was a marker compound specific of ‘Nun 04245 ARA’ (Log FC value = 19.1). Several glycosidic and isomeric forms of apigenin discriminated (i.e., VIP score > 1.1) mainly the cultivars ‘Capriccio’ and ‘Opera’ (Table S3). The cultivar ‘Istar’ was the best source of apigenin 6,8-di-*C-*glucoside (LogFC value = 21.3). Also, seven discriminant compounds were found among sesquiterpene lactones, with mintsulfide (VIP score = 1.35) and epi-antheindurolide A (VIP score = 1.23) possessing the higher discrimination potential. Mintsulfide was a characteristic marker of the cultivar ‘Opera’ (LogFC = 22.1). Epi-antheindurolide A discriminated mainly ‘Capriccio’ (LogFC = 20.4) Finally, the remaining discriminant classes of compounds included amino acids and derivatives, benzenoids, lipid-derivatives, organoheterocyclic compounds, physalins derivatives and other terpenoids (such as mono-, di-, and tri-terpenoids). The L-Histidine (an essential amino acid) was listed as discriminant markers, with a VIP score = 1.19. This compound was annotated in all the artichoke cultivars with different abundance, except for ‘Opal’. ‘Istar’ and ‘Madrigal’ showed the highest LogFC values for this essential amino acid (LogFC = 24.5 and 24.0, respectively). Artichoke leaves are rich in palmitic acid (16:0) and alpha-linolenic acid derivatives (18:3n-3) [38]. Accordingly, we found a good distribution among the discriminant makers of glycerophospholipids, including PE (20:3(8Z,11Z,14Z)/15:0) (potential marker of ‘Madrigal’; Table S3), followed by PGP (16:0/18:0) (potential marker of ‘Nun 04245 ARA’; Table S3). In the secondary metabolites class were included a wide range of chemically diverse and functionally important compounds, like phenolics, alkaloids, and terpenes. Broadly, these secondary metabolites are often produced as an adaptive response to specific stress [39,40,41].

The high diversity of the metabolites among hybrids is likely to correlate with a plant functional diversity. In the future, it will be also interesting to evaluate, also in quantitative terms, if the interaction with environmental factors will produce an alteration of the metabolic profiles that is also strongly influenced by the artichoke variety.

## 3. Materials and Methods

### 3.1. Greenhouse Conditions, Artichoke Cultivars and Experimental Design

The trial was carried out during the autumn-winter growing cycle in a polycarbonate greenhouse at the Experimental Farm ‘Nello Lupori’, University of Tuscia Viterbo, Italy. The seven seed-propagated artichoke (*C. cardunculus* L. subsp. *scolymus* (L.) Hegi) cultivars were: ‘Istar’ and ‘Romolo’ (obtained from La Semiorto Sementi, Sarno, Italy), ‘Capriccio’, ‘Madrigal’, ‘Nun 04245 ARA’, ‘Opal’ and ‘Opera’ (Nunhems Netherlands BV, Haelen, The Netherlands) were sown on September 8, 2016. These cultivars were selected as representative of the main classes of commercial hybrids. With reference to the time of beginning of the main stem elongation and to the color of the outer bracts, the varieties can be described as: ‘Capriccio’: early, violet; ‘Istar’: early, predominantly green; ‘Madrigal’: late, green; ‘Nun 04245 ARA’: early, predominantly violet; ‘Opal’: early, violet; ‘Opera’: early, deep violet; ‘Romolo’: medium-late, predominantly violet. At the two-true leaf stage (September 23, 15 days after sowing; DAS), plants were moved to a floating raft system. The system consisted of 84 polystyrene rafts in heavy-duty plastic tanks with a constant volume (60 L) of fresh nutrient solution. The dissolved oxygen concentration in the nutrient solution was always higher than 6 mg L^−1^. The planting density was 531 plants per square meter, according to that used in professional horticulture. Globe artichoke cultivars were fertigated since 15 DAS with a modified Hoagland nutrient solution with a pH of 6.0 and an electric conductivity of 2 dS m^−1^. Concentrations of macro and micronutrients in the nutrient solution were: 13 mmol L^−1^ nitrate, 1 mmol L^−1^ ammonium, 1.75 mmol L^−1^ sulphur, 1.5 mmol L^−1^ phosphorus, 5 mmol L^−1^ potassium, 4.5 mmol L^−1^ calcium, 2 mmol L^−1^ magnesium, 20 μmol L^−1^ iron, 9 μmol L^−1^ manganese, 0.3 μmol L^−1^ copper, 1.6 μmol L^−1^ zinc, 20 μmol L^−1^ boron and 0.3 μmol L^−1^ and molybdenum. The seven treatments (i.e., artichoke cultivars) were arranged in a randomized complete block design with three replicates, which consisted of one independent tank containing 84 plants each, for a total of 1764 plants divided into 21 experimental units.

### 3.2. Leaf Biomass Determination and Mineral Analysis

The artichoke plants were harvested when the plant height reached approximately 25 cm. The artichoke leaf tissue of each cultivar was dried in a forced-air oven at 70 °C for 3 days until constant weight. Leaf dry matter percentage was also calculated using the following formula: DM (%) = 100 × Dry weight/Fresh weight. The artichoke leaf tissue of the seven seed-propagated cultivars were analyzed for the following macro elements: nitrogen, phosphorus, potassium, calcium and magnesium. One gram of basil ground material was used for the determination of total nitrogen using the Kjeldahl method [42]. Ground material (0.25 g) was analyzed by ion chromatography (ICS-3000, Dionex, Sunnyvale, CA, USA) to determine the mineral content (P, K, Ca, and Mg) according to the method proposed by Rouphael et al. [11]. The results were expressed as mg g^−1^ dry weight (dw).

### 3.3. Extraction of Phytochemicals and UHPLC-QTOF Profiling of the Different Artichoke Cultivars

A homogenizer-assisted-procedure was used to extract metabolites from leaves. Three replicates (1.0 g) of dried leaf tissue were extracted in 10 mL of 80% methanol acidified with 0.1% formic acid, by using an ULTRA-TURRAX (Ika T25, Staufen, Germany) at 5000 *g* for 3 min. The extracts were then centrifuged (Eppendorf 5810R, Hamburg, Germany) at 10,000× *g* for 10 min at 4 °C. The resulting solutions were stored in amber vials until further analyses.

The phytochemical composition was investigated through untargeted metabolomics based on ultra-high-pressure liquid chromatography coupled to a quadrupole-time-of-flight mass spectrometer (UHPLC-ESI/QTOF-MS), using a 1290 series liquid chromatograph coupled to a G6550 iFunnel mass spectrometer through a Dual Electrospray Jet Stream ionization system (all from Agilent Technologies, Santa Clara, CA, USA). The mass spectrometer was set to operate in full-scan mode and positive polarity, acquiring ions in the range of 100–1200 *m/z*. The analytical conditions for the analysis of bioactive compounds in these food matrices were optimized in previous experiments [6]. Briefly, the chromatographic separation was achieved using a Knauer Blue Orchid C18 column (100 mm × 2 mm i.d., 1.8 μm) and a mixture of water and acetonitrile (both LC-MS grade, VWR, Milan, Italy) as mobile phase. Formic acid (0.1%) was added to both solvents as a phase modifier. A gradient separation, from 6% acetonitrile to 94% acetonitrile within 33 min, and a flow rate of 220 μL min^−1^ were used for chromatographic separation. The injection sequence was randomized, with three replicates (6 μL injection volume) for each sample, and a quality control (QC; prepared from the same aliquot of each sample) was run every 9 samples. Raw data were processed using the Agilent Profinder B.06 software, according to the ‘find-by-formula’ algorithm. Monoisotopic accurate mass and the entire isotope pattern (isotopic spacing and isotopic ratio) were used to achieve high confidence in annotation. A custom database was built and used as reference, combining annotations on phenolic compounds (from Phenol-Explorer), terpenoids and those reported on FoodDB. The strategy adopted allowed a Level 2 of identification (i.e., putatively annotated compounds), with reference to the COSMOS Metabolomics Standards Initiative [43,44]. Annotated compounds were retained when passing post-acquisition filters (mass accuracy tolerance 5 ppm, retention time shift < 0.1 min, presence in 100% of replications within one treatment) and threshold (min abundance 10.000 units), possessing a relative standard deviation of <30% in QCs, with plausible chromatographic peak features. To provide semi-quantitative information, cumulative intensity for phenolic compounds (grouped into homogenous classes) and sesquiterpene lactones were converted in mg phenolic equivalents g^−1^, through calibration curves obtained from pure standard compounds (Extrasynthese, Lyon, France), each having a purity ≥98%. Standard were: sesamin (lignans), chlorogenic acid (phenolic acids), cyanidin (anthocyanins), tyrosol (phenyl alcohols and low-molecular-weight phenolics), catechin (flavan-3-ols), quercetin (flavonols), luteolin (flavones and the remaining flavonoids) and cynaropicrin (sesquiterpene lactones) were used. Calibration curves were built using a linear fitting (un-weighted and not forced to axis-origin) in the 0.05–500 mg L^−1^ range; a minimum correlation coefficient (R^2^) of 0.98 was used as the acceptable threshold for the calibration curves.

### 3.4. Statistical Analysis

Statistical analysis of the leaf biomass production, mineral profile, and phytochemical composition was performed using a one-way analysis of variance (ANOVA), followed by post hoc analysis with the Duncan’s multiple range-test (α < 0.05), using SPSS Statistics v. 26.0 software (IBM, Armonk, NY, USA). The F-values and degree of freedom (df) of the statistical analyses are provided in Table S4. Metabolomics-based data on the phytochemical profiles were interpreted using the software Mass Profiler Professional B.12.06 (Agilent Technologies). The data treatment consisted of filtering by abundance and by frequency, followed by a normalization at the 75th percentile and baselining to the corresponding median in the dataset. Unsupervised hierarchical cluster analysis (HCA) using ‘Wards’ as linkage rule with Squared Euclidean distance for dissimilarity measure was performed. The metabolomic dataset was exported into SIMCA 13 (Umetrics, Malmoe, Sweden), UV scaled and elaborated for both principal component analysis (PCA) and Orthogonal Partial Least Squares-Discriminant Analysis (OPLS-DA) supervised modelling. For the latter, variation between the observation groups was separated into predictive (technical variation) and orthogonal (biological variation) components. The OPLS-DA model was checked for outliers (by using Hotelling’s T-squared distribution) and then cross validated by means CV-ANOVA (*p* < 0.01). A permutation test was performed to exclude over fitting after inspecting model parameters (goodness-of-fit R^2^Y and goodness-of-prediction Q^2^Y). The variable importance in projection (VIP) method followed by a Fold-Change analysis (cut-off > 2) was then used to evaluate the marker compounds that better discriminate the artichoke cultivars. In this regard, a VIP score greater than 1.1 was used to select relevant compounds.

## 4. Conclusions

Our results allowed for the identification of a broad diversity of polyphenols and sesquiterpene lactones. They indicated that artichoke leaves growing in advanced horticultural systems (e.g., high-density, short cycle, hydroponics, protected culture) are a rich and valuable source of bioactive molecules. The here analyzed modern hybrids have distinctive metabolomics profiles, with flavonoids being, overall, the most represented class of phenolics, and hydroxycinnamic acids the less abundant. Differences in the mineral content were limited. In our experimental system, the phenotypic variation is mainly attributable to genetic factors and, under this perspective, the high-density floating raft technique proved to be a suitable system to yield cultivar-specific metabolic profiles. Nonetheless, the interpretation of the artichoke metabolomics diversity, and more notably, of its quantitative intra-specific variation, cannot exclusively rely on adaptive defensive mechanisms and ecological interactions. It should more likely result from a stabilizing selection that fixed, and probably partitioned, pre-existing differences in breeding populations. For the analyzed cultivars, our findings imply that ‘Opal’ and ‘Istar’ are the most promising hybrids, producing a good biomass and being the richest sources of phenolics. These included a range of functional phytochemicals of known interest for the food, therapeutic, cosmetics, and nutraceutical sectors.

## Figures and Tables

**Table 1:** Total nitrogen (N), phosphorus (P), potassium (K), calcium (Ca) and magnesium (Mg) concentrations of seven artichoke cultivars grown in a floating raft culture. For each mineral element, different letters indicate significant differences (*p* ≤ 0.05) according to Duncan’s multiple-range test.

Cultivars	N	P	K	Ca	Mg
	(mg g^−1^ dw)	(mg g^−1^ dw)	(mg g^−1^ dw)	(mg g^−1^ dw)	(mg g^−1^ dw)
Capriccio	50.07 ± 0.85	7.12 ± 0.29 ^ab^	72.95 ± 1.35 ^c^	9.52 ± 0.27	3.47 ± 0.38 ^c^
Istar	50.23 ± 2.05	5.76 ± 0.20 ^d^	76.06 ± 2.46 ^bc^	10.33 ± 0.35	4.35 ± 0.41 ^a^
Madrigal	47.19 ± 2.61	7.80 ± 0.57 ^a^	80.00 ± 2.46 ^a^	9.96 ± 0.72	4.17 ± 0.25 ^ab^
Nun 04245 ARA	47.08 ± 2.11	5.32 ± 0.69 ^d^	73.77 ± 1.41 ^c^	9.58 ± 0.70	3.82 ± 0.36 ^abc^
Opal	47.27 ± 2.22	6.68 ± 0.20 ^bc^	68.92 ± 2.05 ^d^	10.43 ± 0.78	3.91 ± 0.04 ^abc^
Opera	47.31 ± 0.47	6.86 ± 0.38 ^b^	78.75 ± 1.74 ^ab^	9.93 ± 0.71	4.03 ± 0.13 ^ab^
Romolo	45.74 ± 1.79	5.89 ± 0.63 ^cd^	82.25 ± 2.97 ^a^	9.16 ± 0.16	3.79 ± 0.07 ^bc^
Significance	ns	***	***	ns	*

ns, *, ***: Not significant, or significant at *p* ≤ 0.05, and 0.001, respectively. All data are expressed as mean ± standard deviation (*n* = 3).

**Table 2:** Semi-quantitative analysis of the main phenolic classes and sesquiterpene lactones (STL) characterizing the different artichoke cultivars. TPC: total phenolic content. Results are expressed as mg phenolic equivalents g^−1^ fresh weight (fw) ± standard deviation (*n* = 3). For each row, the superscript letters indicate statistically homogenous groups according to ANOVA (*p* < 0.05, Duncan’s post hoc test).

	Capriccio	Istar	Madrigal	Nun 04245 ARA	Opal	Opera	Romolo
Anthocyanins	0.018 ± 0.001 ^b^	0.036 ± 0.010 ^c^	0.006 ± 0.003 ^a^	0.007 ± 0.002 ^a^	0.008 ± 0.001 ^a^	0.016 ± 0.001 ^b^	0.017 ± 0.001 ^b^
Phenyl Alcohols	1.705 ± 0.060 ^ab^	3.334 ± 0.214 ^f^	2.611 ± 0.276 ^d^	2.264 ± 0.122 ^cd^	3.011 ± 0.160 ^e^	1.450 ± 0.341 ^a^	1.894 ± 0.192 ^bc^
Flavan-3-ols	0.034 ± 0.005 ^a^	0.064 ± 0.003 ^bc^	0.048 ± 0.006 ^ab^	0.070 ± 0.019 ^bc^	0.088 ± 0.016 ^c^	0.086 ± 0.019 ^c^	0.095 ± 0.023 ^cd^
Flavones	1.244 ± 0.255 ^c^	0.545 ± 0.223 ^ab^	0.554 ± 0.154 ^ab^	0.413 ± 0.077 ^a^	0.692 ± 0.090 ^ab^	0.566 ± 0.086 ^ab^	0.768 ± 0.013 ^b^
Flavonols	0.028 ± 0.007 ^c^	0.089 ± 0.014 ^d^	0.008 ± 0.001 ^ab^	0.006 ± 0.003 ^a^	0.011 ± 0.003 ^ab^	0.029 ± 0.002 ^c^	0.019 ± 0.002 ^bc^
Phenolic Acids	0.296 ± 0.050 ^a^	0.304 ± 0.081 ^a^	0.437 ± 0.020 ^b^	0.436 ± 0.070 ^b^	0.410 ± 0.012 ^ab^	0.361 ± 0.109 ^ab^	0.314 ± 0.038 ^a^
Lignans	0.433 ± 0.076	0.324 ± 0.070	0.392 ± 0.099	0.461 ± 0.085	0.486 ± 0.130	0.506 ± 0.184	0.549 ± 0.102
TPC	3.76	4.69	4.06	3.66	4.71	3.01	3.66
STL	2.559 ± 0.307 ^c^	2.289 ± 0.3789 ^bc^	1.979 ± 0.354 ^ab^	1.616 ± 0.199 ^a^	1.933 ± 0.379 ^ab^	2.426 ± 0.230 ^bc^	1.980 ± 0.119 ^ab^

**Table 3:** VIP marker compounds having the highest discrimination potential when considering the seven artichoke cultivars under investigation. SE = standard error.

Chemical Class	VIP Marker (from OPLS-DA Modelling)	VIP Score	SE
Alkaloids	Harmalol	1.10	0.24
Alkylphenols	4-Vinylguaiacol	1.11	0.48
	4-Ethylcatechol	1.25	0.40
	3/4-Methylcatechol	1.11	0.70
Amino Acids and Peptides	*N*-Succinyl-2-amino-6-ketopimelate	1.22	0.34
	l-Histidine	1.19	0.58
	indole-3-acetyl-valine	1.17	0.86
Benzenoids	2,4-Dinitrophenylhydrazone/Hydroxyanigorufone/Irenolone	1.21	0.33
	1/2-Phenylethyl formate/Ethyl benzoate/3-Methylphenylacetic acid	1.14	0.48
Carboxylic Acids and Derivatives	*N*-Acetylhistamine	1.26	1.15
Flavonoids	Gallocatechin/Epigallocatechin	1.22	1.27
	Eriodictyol/2-Hydroxynaringenin	1.22	1.26
	Eriocitrin/Neoeriocitrin	1.21	0.89
	Chrysoeriol 7-*O*-apiosyl-glucoside/Apigenin 6,8-di-*C*-glucoside/Luteolin 7-*O*-rutinoside	1.16	0.88
	Apigenin 6-*C*-glucoside 8-*C*-riboside/Apigenin 6-*C*-glucoside 8-*C*-arabinoside/Kaempferol 3-rhamnoside 4′-xyloside/Apigenin 7-apiosyl-glucoside/Apigenin 6-C-arabinoside 8-*C*-glucoside/Apigenin 6-*C*-glucosyl-*O*-arabinoside/Apigenin 6,8-*C*-galactoside-*C*-arabinoside/Apigenin 7-*O*-apiosyl-glucoside/Apigenin 6,8-*C*-arabinoside-*C*-glucoside	1.12	0.60
	Kaempferol 3-*O*-rutinoside/Kaempferol 3-*O*-galactoside 7-*O*-rhamnoside	1.16	0.88
	Kaempferol 3-rhamnoside 7-xyloside/Kaempferol 3-arabinofuranoside 7-rhamnofuranoside	1.12	0.60
	3′,4′,5,7-Tetrahydroxyisoflavanone	1.22	1.26
	Petunidin 3-*O*-rutinoside/Cyanidin 3-*O*-rutinoside/Pelargonidin 3-*O*-sophoroside	1.16	0.88
	6′-Hydroxyangolensin/5′-Methoxy-*O*-desmethylangolensin	1.15	0.77
	Norartocarpanone/Aromadendrin/Dalbergioidin/Dihydrokaempferol	1.22	1.26
	Leucocyanidin/4-Gallocatechol	1.22	1.27
	Vicenin 1-3/Apiin/Vitexin 2″-xyloside/Corymboside/Schaftoside/Isoschaftoside/Neoschaftoside/Isovitexin 2″-arabinoside/Kaempferol 3-*O*-arabinosyl 7-*O-*rhamnoside	1.12	0.60
Lignans	Schisanhenol	1.24	0.72
	10-Methoxyyangonin	1.15	0.77
Lipids and Lipid-Like Molecules	3-Hydroxyphenyl-valeric acid	1.43	0.54
	Methylglutarylcarnitine	1.14	0.23
	Aeglin	1.12	0.30
	3-oxo-2-(*cis*-2′-pentenyl)-cyclopentane-1-octanoate	1.12	0.55
	Docosanamide	1.10	0.37
	PE(20:3(8Z,11Z,14Z)/15:0)	1.14	0.39
Methoxyphenols	Guaiacol	1.11	0.70
	6-Gingesulfonic acid	1.38	0.52
	5-Ethenyl-2-methoxyphenol/2-Methoxy-4-vinylphenol	1.14	0.48
OrganoheteroCyclic Compounds	2,3-Dihydro-6-methyl-5-(5-methyl-2-furanyl)-1H-pyrrolizine	1.22	0.33
	Cytokinin B	1.15	0.44
	2-Ethyl-4-(2-furanyl)-2-propenal/2-(2-Furanyl)-3-methyl-2-butenal/3′-Methoxyacetophenone/2′-Methoxyacetophenone/4′-Methoxyacetophenone/2′-Hydroxy-5′-methylacetophenone/2-Acetyl-1-hydroxy-4-methylbenzene/Acetoanisole	1.14	0.48
Phenol Ethers	4-Ethoxybenzaldehyde	1.14	0.50
Phenolic Acids	4-Ethylbenzoic acid/3,4-Dimethylbenzoic acid	1.14	0.49
Phenolic Glycosides	Phlorin	1.15	0.83
Phenylacetamides	Atenolol	1.11	0.64
Phenylpropanoic Acids	2-Phenylpropionate/3-Phenylpropanoic acid	1.14	0.48
Physalins and Derivatives	Isophysalin B/Physalin B/Physalin C/25,27-Dihydro-4,7-didehydro-7-deoxyphysalin A	1.29	0.44
Plant Hormones	Gibberellin A1 glucosyl ester	1.12	0.30
Sesquiterpenoids	Mintsulfide	1.35	0.83
	epi-Antheindurolide A	1.23	0.64
	Tataroside	1.15	0.34
	Antheindurolide A	1.12	0.21
	Lactucain B	1.11	0.70
Diterpenoids	Armillane	1.24	0.72
	(*R*)-3,4-Dihydro-2-methyl-2-(4,8,12-trimethyl-3,7,11-tridecatrienyl)-2*H*-1-benzopyran-6-ol	1.17	0.33
Monoterpenoids	Tsugaric acid B	1.14	0.82
Triterpenoids	Ganoderic acid H	1.11	1.26
Phenyl Alcohols	Tyrosol	1.25	0.40

## Supplementary Materials

Table S1: Metabolomic dataset from UHPLC-QTOF-mass spectrometry containing all the annotated classified compounds together with their SMILES, PubChem IDs, InChIKey codes, and composite mass spectra (i.e., mass & abundances combination). The relative abundance values for Quality Control sample replicates are also provided. The values equal to 1 into the dataset correspond to not identified compounds. Besides, representative total ion chromatogram (TIC) plots for each sample extract are also provided. Table S2: Cross-Validation parameters (CV-ANOVA and Hotelling’s T-squared distribution) and permutation testing (N = 200) following OPLS-DA supervised modelling. Table S3: LogFC values deriving from Fold-Change (FC) analysis, considering the VIP discriminant compounds from OPLS-DA supervised modelling. Table S4: One-Way ANOVA Sums of Squares, Mean Squares, and F-test.
